# Prioritizing Equity: Exploring Conditions Impacting Community Coalition Efforts

**DOI:** 10.1089/heq.2019.0061

**Published:** 2019-08-21

**Authors:** Ariel M. Domlyn, Shemekka Coleman

**Affiliations:** ^1^ Department of Psychology, University of South Carolina, Columbia, South Carolina.; ^2^ Institute for Healthcare Improvement, Cambridge, Massachusetts.

**Keywords:** community coalitions, qualitative comparative analysis, program evaluation, health inequity

## Abstract

**Purpose:** There is a critical push toward addressing equity in health care. Community coalitions are uniquely situated to heed this call by tackling issues of equity and well-being that are the most relevant for their local context. This article analyzes internal and external contextual factors that may affect coalitions' prioritization of equity.

**Methods:** Data were collected from 18 coalitions participating in a national, U.S.-based initiative aimed at strengthening community coalition work through the principles of equity and inclusion. A hybrid qualitative–quantitative method (qualitative comparative analysis [QCA]) was conducted using the direct method of calibration and fuzzy set QCA and to obtain casual sufficiency results.

**Results:** Coalitions located in states that did not expand Medicaid after the Affordable Care Act were most likely to prioritize equity, as were coalitions who were both working with marginalized populations and had low organizational readiness for the initiative. However, only one case demonstrated the latter causal solution; the former accounts for greater coverage of the outcome.

**Conclusion:** This study illustrates the use of QCA for evaluation and underscores the critical role of contextual factors for affecting meaningful community-level change. Coalitions are willing and able to prioritize tackling health inequities across settings, but those in settings with low state-level support may be more likely to emphasize inequities in their work.

## Introduction

In the United States, health inequities lead to avoidable differences in health outcomes.^1^ The effects of health disparities are devastating to the marginalized people who are the most affected and are indicative of inequities in access, treatment, and prevention of disease. Despite federal efforts to target this issue, limited progress has been made for reducing health disparities for any racial and ethnic groups.^2^

Although little progress has been made through national action, community coalitions offer a front-line offense for tackling this issue. These coalitions, composed of organizations dedicated to filling specific needs in the community, can address inequities and racism via targeted local action.^1^ This is important given the relevance of geographic setting for shaping health.^3^ Large-scale programs supporting and elevating coalitions' efforts play an important role in achieving community-level change. This article will discuss an analysis conducted within a coalition support program to better understand factors that influence whether coalitions prioritize health equity.

Communities are influenced by their ecological context and can be conceptualized as a “mediating force linking the micro level of the individual and the macroeconomic, political and cultural structures that shape society.”^4^ Drawing from the socioecological model, this study focused on the interplay of social climate variables across multiple levels.^5^ Researchers on health disparities have noted the dearth of studies that investigate the multiple ecological levels (e.g., organizations, communities, systems) affecting inequity.^6^ To best conceptualize the factors that could influence a coalition's choice to prioritize equity, this study focused on variables related to the organizational, locality, and macrosystemic context. Each factor considered potentially relevant for the coalition was assessed through the lens of the coalition's perspective. Thus, organizational factors include the readiness of the coalition to engage in community-level changes. Locality factors include the salient local issues that each coalition is targeting. Macrosystemic factors include the political climate in which the community is nested. The primary question that this study intended to answer is: Among coalitions participating in a health transformation initiative, across these ecological levels, what affects whether they prioritize issues of equity?

## Methods

### Sample

The cohort for this study was composed of 18 community health coalitions around the country involved in SCALE, an intensive program aiming at building the readiness and capacity of coalitions to create a Culture of Health, of which health equity is a key focus.^7^ Coalitions were chosen for this program based on demonstrated efficacy in community-level change and goals aligned with the vision of becoming a Community of Solutions.^7^ The communities represent a broad swath of the country, ranging from urban to rural, varying in population size, and each targeting a different segment of their community (Table 1). Each community consists of a multi-stakeholder coalition of organizations targeting issues of health affecting their area. Examples of organizations within a coalition include (but are not limited to): departments of public health, recovery houses, women's shelters, community mental health clinics, hospital administrations, and homeless shelters. At the time of data collection, coalitions had been involved in the SCALE initiative for 2 years.

**Table 1.** Community Characteristics

Coalition	State	Urban vs. rural	Community size	Target health issue
A	NM	Urban, rural, other	500,001 and higher	Opioid use and child well-being
B	OH	Urban	5001–50,000	Youth, healthy eating, nutrition, obesity
C	NY	Rural	50,001–100,000	Childhood obesity
D	UT	Urban, suburban	50,001–100,000	Black/African refugees
E	WV	Rural	5001–50,000	Healthy eating/food access, chronic disease, physical activity
F	AZ	Urban	500,001 and higher	Obesity, diabetes, lung cancer, cardiovascular disease, access to care, healthy food, early childhood
G	SC	Suburban, rural	5001–50,000	High school health, tobacco use
H	WY	Rural	50,001–100,000	Youth homelessness
J	NH	Rural	50,001–100,000	Healthy schools, workplaces, and neighborhoods
K	IL	Urban	5001–50,000	Obesity and food access
L	NC	Urban	5001–50,000	Youth and family health and well-being
M	CA	Urban, suburban	500,001 and higher	Youth, healthy eating, food access
N	CA	Urban	5001–50,000	Homelessness among women
P	OH	Urban, suburban, rural	500,001 and higher	Reduce infant mortality
Q	CA	Urban	5001–50,000	Parks and safety
R	MA	Urban	500,001 and higher	Children's well-being in primarily minority neighborhoods
S	ME	Other	5001–50,000	Food access
T	OK	Urban, rural	500,001 and higher	Food access

### Measures

The outcome variable for this study was the degree to which coalitions prioritized health equity. Building on 2 years of learning and growing with SCALE, in late 2017, each coalition crafted their first action plans for spreading the initiative's health transformation model to other organizations, coalitions, and communities. As a tangible marker of the intention to realize community-based health transformation, those action plans provide the outcome data.

Considering factors that are potentially relevant for prioritizing issues of equity, this study captured variables that are relevant for the coalition and the context. For the coalition, organizational readiness is critical for undertaking any change.^8^ Accounting for the different populations served, data were gathered on the population that each coalition focused on (e.g., youth, racial or ethnic minorities, homelessness) and the target issues that each coalition was tackling (e.g., substance use or prevention, nutrition and food access). A measure of current functioning on health equity was also included; this baseline accounts for whether coalitions were more explicit from the start on prioritizing equity.

To capture macropolicy and climate variables, overall political climate is a relevant factor for the achievement of health equity.^9^ As a proxy of political climate, the decision of each state to expand Medicaid as optioned by the Affordable Care Act (ACA) was included as a condition. Lack of health insurance is a key component allowing inequities to persist, and the expansion of Medicaid was expected to expand coverage to an additional 12 million people nationwide by 2017.^10^ This is also a rough indicator of overall racial climate in each state, as research suggests that the decision to expand Medicaid was highly racialized.^11^

Data for this study were pulled from four sources, each of which initially served the purpose of either gathering information about the coalition or monitoring progress in the initiative. These sources included (1) coalitions' application for the initiative, which included information in Table 1, whether their state had chosen to expand Medicaid, and the primary health issues and populations targeted by their coalition. (2) Readiness Diagnostic Tool (RDT), an assessment of three components and 18 subcomponents of organizational readiness.^12^ The RDT self-report measures readiness for health transformation, a key component of this transformation being health equity across their community. An average of each coalitions' responses across all items composed the readiness score used for this analysis. (3) Community Transformation Map (CTM) is a SCALE-specific assessment and planning tool intended to capture all aspects of community health transformation. Based on innovation-configuration maps,^13^ this self-reported scale provides both an assessment of current functioning as well as a map of what the next level of achievement should look like for optimal implementation. (4) Coalitions' action plans for 2018 constitute a requirement of the initiative that addressed their intended aims as well as achieving the goals of SCALE. These plans were then assessed for quality by members of the SCALE evaluation team. A rubric was created to evaluate the degree to which each coalition explicitly prioritized issues of equity in their plans. The resulting score is the outcome for this study. Levels of equity prioritization were determined on four criteria: whether the coalition listed equity items among their top five goals, whether their strategy for achieving these goals explicitly addresses equity, whether their quarterly goals link to racism or health equity, and whether their intended capacity assessment of their community addresses equity. Each criterion was graded on a scale and weighted to create an equity prioritization score for each coalition. Stated goals are not necessarily indicative of ability to achieve these aims, whereas research on organizational goal-setting behavior indicates that a shared vision and growth goals are indicative of future development^14^ and serve as primers for eventual goal attainment.^15^ Coding this condition, it was considered whether there was a theoretical fit between rating a coalitions' explicit aims, versus that which might be implied in their work given the context. Existing literature suggests that specificity of goals is a key component of performance and attainment,^16^ therefore the choice to focus on their explicit aims is considered valid.

### Analysis

Based in set theory and the idea of complex causality, qualitative comparative analysis (QCA) is a hybrid qualitative–quantitative method used to explore concept formation, typologies, or reveal patterns that are indicative of causal explanations for events. QCA determines the necessary and sufficient conditions—and combinations of conditions—that result in a specified outcome. It relies on knowledge of cases that can be coded on each condition and the outcome factor.^17^ It is theorized to be analytically superior to many statistical techniques^18^ and is ideal for research of medium *N* data sets (10–50 cases), though it can be used on larger data sets. Using Boolean algebra, cases are systematically compared to identify relevant patterns within sets, determining the conditions and combinations of conditions that satisfy the outcome.^19^ The method has been applied to many contexts, including evaluating program effectiveness^20^ and identifying key conditions for successful implementation in health settings.^21^

QCA is an ideal analytic technique for this study because it incorporates both qualitative and quantitative data, allows for exploratory analysis, and is designed for small data sets. This analysis used a fuzzy set QCA (fsQCA) where the degree to which each set satisfies the outcome or condition is determined on a gradient of membership rather than a binary presence–absence coding scheme.^22^ QCA determines sufficiency, where the outcome always or almost always occurs when the condition is present, indicating that the condition is a subset of the outcome. Sufficiency analyses provide multiple possible outcome “recipes,” from most conservative to a parsimonious combination. Some evidence indicates that the parsimonious solution is most reliable,^23^ therefore this solution will be provided here. These are reported by consistency and coverage scores of sets. Consistency refers to the degree to which the data support the presence of a set relationship between the condition(s) and outcome, whereas coverage explains the degree to which condition(s) explain the outcome. A minimum consistency threshold is at least 0.75,^24^ with 1 indicating that the condition and outcome are always connected. Calibration of conditions was conducted by using the direct method of fuzzy set calibration.^25^

## Results

Analyzing sufficiency of both single conditions and a combination of conditions, two causal recipes were shown to result in the outcome and are presented in Table 2. Together, the two combinations often (consistency=0.86) cover 54% of the outcome. This indicates that there are other conditions accounting for when coalitions prioritize equity, but these two combinations consistently cover a large portion. These solutions are equivalent, where both lead to the outcome. In Table 2, raw coverage is the part of the outcome explained by this solution whereas unique coverage refers to the part of the outcome explained by only that solution. The equivalence of these outputs means that the sets analyzed did not overlap on the combinations analyzed, and either leads to the outcome.

**Table 2.** Parsimonious Sufficiency Solution

Combination	Raw coverage	Unique coverage	Consistency	*N*
State did not expand Medicaid	0.46	0.45	0.86	6
Coalition is working with minority or homeless populations *AND* they had low readiness scores	0.09	0.08	0.89	1
Solution overall: coverage=0.54, consistency=0.86				

The first sufficiency recipe indicates that coalitions located in states that did not opt to expand Medicaid after the ACA often (consistency=0.86) prioritized equity in their plans. These coalitions (*N*=6) are working to affect health in states demonstrating minimal support for low-income citizens who could benefit from the expansion of Medicaid. The second sufficiency recipe suggests that coalitions who are working with minority or homeless populations and have low readiness for becoming a Community of Solutions, measured via RDT scores, often (consistency=0.89) prioritized equity in their plans. In contrast to the first sufficiency solution, this coalition may be motivated to explicitly target equity in their planning because of the populations they are working with but they clearly have greater issues to overcome in motivation and capacity. However, only one coalition is implicated in this second causal recipe and it accounts for a very small amount of the outcome (unique coverage=0.08), indicating that it is not a significant solution.

Case groups in the first solution include coalitions D, G, H, L, S, and T. These coalitions are in Utah, South Carolina, Wyoming, North Carolina, Maine, and Oklahoma. Some are centered in urban areas (D, L, T) and others in suburban or rural (G, H, S). They vary in the populations they seek to affect, including minorities, youth, and those experiencing homelessness. Some also look to improve food access and mitigate substance use in their communities. The coalitions in this solution vary by geography, demographics, and aims. However, they are similar in that each are located in states that declined federal funds to expand Medicaid. This rough indicator of state health politics suggests that each of these coalitions is fighting against additional systemic barriers to achieving health equity. Their macropolitical context suggests that these coalitions may prioritize health equity in their plans because they perceive the necessity to act locally in lieu of state support.

## Discussion

This study has several strengths. First, it was conducted using measures that are specific to the program being evaluated. Although this limits generalizability of these findings, it also ensures that each data point is valid for this setting. Multiple data sources were used to calibrate conditions, and separate teams coded the conditions and the outcome. In addition, the condition coders were blinded to the outcome. Another benefit is that the study sample includes all current SCALE coalitions and is not limited by selection bias. SCALE coalitions are diverse in size, location, mission, and population served. This complexity made the selection of QCA ideal because it is a method developed to study complex phenomena with limited sample sizes.

### Implications

Results of this study hold implications for the focus of programs centered on health transformation. Among coalitions that did not adequately include equity in their action plans, all are in states that did choose to expand Medicaid. This underscores the significance of the macropolitical context for achievements in health equity. In states that lack the will to improve health care coverage for low-income families, communities must take action to mediate the effect of political and cultural structures on individual health.^4^ The cases in this study suggest that health coalitions heed the call when state support is not present. This is particularly true among those coalitions that may not have already focused their efforts on the most vulnerable in their communities.

#### Programs supporting community coalitions

Results indicate that coalitions in a variety of contexts are willing and able to prioritize taking action to tackle health inequities. An encouraging finding was that coalitions not specifically working with marginalized and underserved populations are working toward prioritizing equity. This shows promise for the development of SCALE and similar programs to support community health improvement. Those that participate in a program such as SCALE may not already be prioritizing equity unless they are already working with marginalized populations. Programs such as these are valuable for ensuring coalitions bring equity and race into their efforts to tackle community health. Knowing that coalitions are likely to prioritize equity if they are located within a less supportive state is useful for resource allocation, as motivation to improve equity may be highest among these communities. Funders may consider including state politics as part of their partner engagement selection guidelines.

#### Evaluation

This study illustrates the incorporation of factors across systemic levels to evaluate outcomes, which should be adopted by evaluators to better understand the interplay of both internal and external factors affecting program success. We also demonstrated the use of a hybrid qualitative–quantitative method in evaluating community coalitions. Few studies have applied this method to community coalitions (see Kane et al.,^26^ for closest example), and this is a relatively novel method in the field of evaluation but is growing in interest.^27^ Future evaluation work should incorporate QCA to understand conditions influencing whether a programmatic priority is adopted. This could lead to a refinement of the method for the evaluation professional.

### Limitations

Conclusions drawn from this study are subject to several points of caution. First, the conditions were chosen by the researcher (a member of the SCALE evaluation team), not the SCALE implementation team nor coalitions. Although data selection and coding were based in theory, a practitioner's perspective would have provided additional information about the program and data. It is also notable that the outcome condition was coded based on an aggregate of equity-related data points. Individual items may better capture the salience of equity in these coalitions and should be subject to further inquiry. Finally, this study did not measure coalitions' actions nor achievements in equity. Instead, this focused on their intentions. SCALE is ongoing and the spread of the Community of Solutions model is just beginning. A follow-up analysis will reveal whether these conditions are relevant for measuring both planning to address equity and enacted improvements in community health inequities.

## Conclusion

Equity is a critical issue for furthering health care efforts in the United States and mitigating unjust systemic issues that allow inequities to persist. Ambitious initiatives such as SCALE boast promising interim results toward fostering a nationwide Culture of Health^7^ by reaching communities in the greatest need of additional resources and works to target critical issues of health and well-being. By using a relatively novel method in the field of evaluation, this study offers a snapshot of factors across socioecological levels that may influence the prioritization of equity.

## Abbreviations Used

ACA: Affordable Care Act
fsQCA: fuzzy set QCA
QCA: qualitative comparative analysis
RDT: Readiness Diagnostic Tool
